# Estimating Genetic Analysis Using Half Diallel Cross Underlying Kenaf (*Hibiscus cannabinus* L.) Fibre Yield in Tropical Climates

**DOI:** 10.1155/2022/1532987

**Published:** 2022-10-19

**Authors:** Md Al-Mamun, Mohd Y. Rafii, Azizah binti Misran, Zulkarami Berahim, Zaiton Ahmad, Md Mahmudul Hasan Khan, Yusuff Oladosu

**Affiliations:** ^1^Laboratory of Climate-Smart Food Crop Production, Institute of Tropical Agriculture and Food Security (ITAFoS), Universiti Putra Malaysia (UPM), 43400 Serdang, Selangor, Malaysia; ^2^Bangladesh Jute Research Institute (BJRI), Dhaka 1207, Bangladesh; ^3^Department of Crop Science, Faculty of Agriculture, Universiti Putra Malaysia (UPM), 43400 Serdang, Selangor, Malaysia; ^4^Agrotechnology and Bioscience Divisions, Malaysian Nuclear Agency, Bangi, 43000 Kajang, Selangor, Malaysia; ^5^Bangladesh Agricultural Research Institute (BARI), Gazipur 1701, Bangladesh

## Abstract

Kenaf (*Hibiscus cannabinus* L.) is a natural fibre crop that can be used for a variety of purposes and has various applications in industry. Despite this, its potential has not been fully exploited because of low yields and a narrow genetic base, limiting hybrids' development. Based on this background, eight kenaf mutants and one commercial cultivar were selected and crossed in a half-diallel for general and specific combining abilities (GCA and SCA) to get the desired results done in this investigation. The 36 hybrid offspring and their parental lines were tested in the field over two environments. Diallel results based on Griffing B method 2 indicated significant differences for all characters studied except for GCA in top diameter and plant height and top diameter SCA, indicating the existence of both additive and nonadditive gene actions for the inheritance of the traits. The amplitude of GCA variation was much higher than that of SCA variation for all parameters except top diameter and node number, showing the additive gene's prevalence and the likelihood of genetic advancement through selection. In both conditions, Hayman and Jinks graphical studies demonstrated that partial dominance controlled various fibre yield component parameters such as plant height, middle diameter, stick weight, and fibre weight. On the other hand, fibre yield and the majority of physical features indicated either dominance or overdominance gene action. Plant height, base diameter, core diameter, middle diameter, fresh stem weight, and stick weight all strongly positively correlated with fibre yield. These traits also had a higher proportion of additive effects, a moderate narrow-sense heritability, and a higher baker ratio, indicating successful indirect selection for fibre yield. The parents P_1_, P_3_, and P_4_ had the most dominant alleles for most of the features, while the parents P_2_, P_7_, and P_9_ had the most recessive alleles. The hybrids P_1_ × P_4_, P_1_ × P_9_, P_2_ × P_3_, P_2_ × P_5_, P_4_ × P_6_, P_4_ × P_7_, P_4_ × P_9_, P_5_ × P_8_, and P_7_ × P_9_ outperformed the parents in terms of heterotic responses and showed that they have a lot of genetic potential for kenaf enhancement in tropical climates.

## 1. Introduction

In recent years, kenaf (*Hibiscus cannabinus* L.) has garnered significant interest as an intriguing multipurpose crop for producing energy, pulp, thermal insulation boards, and fibre-reinforced thermoplastic composites in Mediterranean nations. It has been employed as an alternate raw material for wood, pulp, paper, and textile manufacturing industries. The economic product of kenaf is the stem material, which consists of the outer bark (bast). The bark of the fibre-producing plant's stem is removed as kenaf through a washing and drying procedure [1]. It creates a high-quality pulp that is also suited for making ropes, cordages, sacs, canvases, carpets, etc. In addition, the central section of the plant, known as the core, is abundant in cellulose and hemicellulose. This makes it a potential source for the synthesis of bioethanol; additionally, it can be used as an absorbent material for animal bedding [2]. According to Dempsey, the productivity of a kenaf F_1_ generation is 14 to 43 percent more than the parents [3]. As part of the ASEAN Free Trade Area (AFTA), the Malaysian government encouraged kenaf planting to replace tobacco to reduce tobacco import duties by 2010 [4]. Despite its wide distribution in tropical countries, kenaf productivity in Malaysia is low due to a lack of high-yielding varieties with fibre production of around 5 to 10 tonnes per hectare, and research into developing new kenaf varieties is still lacking [5]. Developing new kenaf varieties that produce high biomass is vital for effective kenaf production [6]. Thus, breeding kenaf in Malaysia is essential for producing high fibre content and adaptable growth in the local climates [5]. In some countries, including China, Russia, and Thailand, kenaf hybrids have been employed for commercial farming contributing to increasing production [7]. Due to its improved fibre quality and resistance to force, hybrid kenaf has gained a lot of interest [8]. In Malaysia, hybrid kenaf technology is considered novel, and more research is required to assess genetics, agronomy, and crop management. A hybrid kenaf breeding programme is required to develop a high-yielding, stable performance hybrid kenaf for the Malaysian environment. The availability of high-quality jute and kenaf seed would help to ensure fibre quality to a large extent [9]. As a result, developing locally adapted hybrid kenaf seeds is a viable alternative for enhancing national kenaf yield and increasing kenaf producer income.

The diallel analysis is a good way to screen parents for hybrid creation when compared to other mating designs. Diallel cross is an appropriate genetic design for obtaining genetic information on desired features in a short period. The ability to forecast the efficiency of selection in a population for improving yield in commercially relevant crops requires understanding the nature of gene action for various traits [10]. To better understand the nature of gene action involved in quantitative trait expression, the diallel mating design has been widely used in both self-pollinated and cross-pollinated species [11]. Plant breeders typically want to know how valuable a group of parents is for breeding. Combining ability analysis is a powerful tool for identifying superior hybrid parents with strong general combining ability (GCA) and progenies with stronger specific combining ability (SCA) [12]. In terms of gene activity in character quantitative inheritance, exploitation, and breeding, it is also useful for quantifying the genetic value of parents and crossings [13].

GCA refers to a parent's average performance over several crosses [14]. On the other hand, SCA refers to a hybrid combination that outperforms or underperforms the parent inbred lines based on their average performance [15]. Cultivar parents with a strong GCA impact have additive gene action but do not always have a good SCA [16]. Meanwhile, genetic studies can benefit from SCA estimate to determine the type of gene action that impacts the phenotypes of interest. Nonadditive gene action is identified by a high SCA [17]. SCA effects were greater than GCA effects when measured in terms of average effects (components), demonstrating the relevance of nonadditive gene action in regulating kenaf yield component expression [18]. SCA and GCA statistics help breeders choose hybrids and parents to produce good offspring [19]. Parents with a high level of GCA generate strong hybrids [20]. The crossing lines with strong GCA are all crucial approaches for improving kenaf production.

The frequency of dominant and recessive genes and the number or cluster of genes affecting the quality of interest can all be used to improve breeding programme planning. Plant breeders use GCA and SCA data to choose appropriate genotypes and generate new high-yielding cultivars [21, 22]. Heritability is a fundamental genetic trait calculated in broad and narrow sense [23]. Selection can increase a trait with a high heritability estimate [2]. It will be extremely beneficial to improve the kenaf crop as a variety in Malaysia's tropical environment by selecting genotypes with unique genetic histories [24]. The main purpose of this study was to find genotypes (parents and offspring) with good combining ability, which could help future kenaf improvement with high fibre production. The goal of this research was to use a diallel analysis to quantify the genetic variation of fibre yield components and examine if different kenaf mutants in tropical climates have different patterns of morpho-physiological parameters combining abilities.

## 2. Materials and Methods

### 2.1. Experimental Location

The research was conducted at Universiti Putra Malaysia, Selangor, Malaysia, at a latitude of 2°59′ N, a longitude of 101°42′ E, and 48 metres above sea level [2]. The field trials took place in two environments: one from June to September 2020 and the other from March to June 2021, both in a humid tropical climate. The maximum and minimum mean monthly temperatures (°C), total rainfall (mm), and relative humidity for the experimental periods are shown in Table 1.

### 2.2. Breeding Materials

Based on morpho molecular characterization [25, 26], eight kenaf mutants and one commercial cultivar were chosen as parents in 9 × 9 half diallel crosses (Table 2). From February to May 2020, the parents were mated in all feasible combinations, except reciprocals, at University Putra Malaysia's Field 10 to produce 36 F_1_ hybrids (Supplementary figure 1).

### 2.3. Development of F_1_ Using Half Diallel Mating Design

Although the genotype clustering differed greatly across the two methodologies, the genotypes were placed in similar groups. Consequently, when choosing parents for hybridized activity, integrating phenotypic and molecular data will provide a more accurate summary. Pure and healthy seeds were transplanted in three batches at 10-day intervals in the trial field to achieve flowering synchronisation. After the fruits had ripened, normal emasculation and pollination methods were used to obtain F_1_ seeds.

### 2.4. Experimental Design and Layout

Mechanical ploughing and laddering were used to prepare the soil for the plant's cultivation. Kenaf seeds from 45 entries comprising nine parents and 36 F_1_s were sown in peat moss soil in germination trays. Twenty-one seedlings were transplanted into a 60 cm × 80 cm plot with a distance of 10 × 40 cm inter- and intrarow spacing after two weeks, to a depth of two to 2.5 cm. There were 135 plots, and the entire plot measured 59 m × 9 m. The experiment was carried out in a Randomised Complete Block Design (RCBD) with three replications using a table of random numbers [27].

### 2.5. Crop Husbandry

Standard kenaf production provided a good crop with the best management. NPK Green (15 : 15 : 15) and NPK Blue (12 : 12 : 17) were applied at 450 kg per hectare shortly after seeding and 40 days after planting. Intercultural activities like weeding, thinning, additional watering, and plant protection measure were carried out as needed throughout the cropping environment. The recommended cultural guidelines for growing a healthy crop were followed as described by Wong et al. [28].

### 2.6. Data Collection

A total of 10 fibre yield components were discovered. For each feature, observations were obtained from 10 randomly selected plants for each genotype for each replication. Plant height, base diameter, core diameter, middle diameter, top diameter, node number, days to 50 percent flowering, fresh stem weight, stick weight, and fibre weight were among the quantitative data collected in the same direction as the procedure of Al-Mamun et al. [25]. Days to 50 percent flowering were confirmed in the field, and remaining attributes were measured 90 days after transplantation in the laboratory.

### 2.7. Statistical Analysis

#### 2.7.1. Performance of Hybrids

An analysis of variance (ANOVA) was used with the PROC GLM function of the Statistical Analysis System (SAS) computer software version 9.4 to determine the significance of variation among genotypes and blocks. A combined ANOVA from 9 × 9 diallel (F_1_ and parents) was performed for each growth environment, eliminating reciprocals as indicated by Steel et al. [29]. The least significant difference (LSD) method was used to compare the mean performance among hybrids and paternal inbred lines.

#### 2.7.2. Combining Ability Analysis

Griffing's and Hayman's techniques [30] were used to analyze the genetics of the diallel population. According to Baker [31], the combining ability ratio was determined. A modified Hayman [32] ANOVA was calculated after Morley Jones [33] adjustment. The genetic system influencing important kenaf features and general and specific combining abilities has been studied.

*(1) Diallel Analysis by Griffing's Method*. Griffing's method 2 (one set of F_1_ progenies and parental lines) was used to analyze the diallel [34]. The GCA of parents and SCA of hybrids were determined following Griffing's method 2 model 1 (fixed effects). According to Zhang and Kang [35], the linear model for data from the combined analysis of performance and environments was as follows:

(1) $$\begin{array}{c} Yijk=\mu +\alpha l+rkl+vij+\left(\alpha v\right)ijl+eijklc, \end{array}$$

where Yijk is the observed value of each experimental unit, *μ* is the mean of the population, *α*l is the environmental effects of *l*^th^ environment, rkl is the replication effect in each environment, vij is the F_1_ hybrid effect, (*α*v) ijl is the interaction effect between F_1_ hybrids and environments, and eijklc is the residual effects.

(2) $$\begin{array}{c} \left(\alpha \text{v}\right)\text{ijl}=\left(\alpha \text{g}\right)\text{il}+\left(\alpha \text{g}\right)\text{jl}+\left(\alpha \text{s}\right)\text{ijl}, \end{array}$$

where (*α*g) il denotes the interaction effect between GCA and environments for the *i*^th^ parent, (*α*g) jl denotes the interaction effect between GCA and environments for the *j*^th^ parent, and (*α*s) ijl denotes the interaction effect between SCA and environments for the ij^th^ F_1_ hybrid.

The combining ability ratio was calculated according to Baker [31] as below:

(3) $$\begin{array}{c} \text{Baker ratio}=\frac{2\text{M}{\text{S}}_{\text{GCA}}}{2\text{M}{\text{S}}_{\text{GCA}}+{\text{MS}}_{\text{SCA}}}, \end{array}$$

where MS_GCA_ and MS_SCA_ are the mean squares for GCA and SCA, respectively.

*(2) Diallel Analysis by Hayman's Approach*. Most plant breeding programs that attempt to improve yield and other related parameters start with parallel analysis. Breeders utilize it the most to determine the utility of varieties as parents and to analyze gene action in diverse qualities [34, 36]. The following are the main features of Hayman's approach: (i) Hayman's ANOVA; (ii) Vr-Wr analysis with graphical representation; and (iii) components of variation and genetic parameters.

*(3) Hayman's ANOVA and Morley Jones Modification*. Hayman [32] analyzed variance for the entire diallel table, expanding on Yates' work [37] in one direction. Reciprocal differences are typically absent; therefore, just one reciprocal cross from each pair is elevated. Morley Jones [33] modified Hayman's technique in response to this situation. The sum of squares correlating to the additive impact (a) in Hayman's model indicates dominance (b_1_), and additional dominance effects that genes can explain with one allele present in only one line (b_2_) (the residual n-1 lines were supposed to bring the same substitute allele), and remaining dominance effects (b_3_) are essentially a straightforward application of least squares suitable constants.

*(4) Vr-Wr Regression Analysis*. The regression coefficient was calculated by using the following formula:

(4) $$\begin{array}{c} \text{Standard error }\left(b\right)={\left[\frac{\left\{\text{Var }\left(\text{Wr}\right)–b\text{ Cov}\left(\text{Wr},\text{Vr}\right)\right\}}{\text{Var}\left(\text{Vr}\right)\left(n-2\right)}\right]}^{1/2}, \end{array}$$

where Vr is the variance of each array, Wr is the covariance between parents and their offspring's, Var (Vr) is the variance of Vr, Var (Wr) is the variance of Wr, and Cov (Wr, Vr) is the covariance between Vr and Wr.

Significant difference of “*b*” from zero and unity was tested as follows:

(5) $$\begin{array}{c} \text{Ho}:b=0=\frac{\left(b–0\right)}{\text{SE}\left(b\right)},\\ \text{Ho}:b=1=\frac{\left(1–b\right)}{\text{SE}\left(b\right)}. \end{array}$$

These results were compared to the “*t*” values for (*n* − 2) degrees of freedom.

The Vr-Wr analysis shows the significant variation of the regression coefficient (*b*) from unity. The absence of nonallelic interaction is demonstrated if the regression coefficient does not significantly depart from unity. The “*t*^2^” test for the uniformity of Vr-Wr values was used to verify the validity of diallel theory assumptions.

(6) $$\begin{array}{c} t^{2}=\frac{\left(n-2\right)}{4}\times \frac{{\left(\text{VarVr}–\text{VarWr}\right)}^{2}}{\text{VarVr}\times \text{VarWr}–{\text{Cov}}^{2}\left(\text{Vr},\text{Wr}\right)}. \end{array}$$

*(5) Components of Variation and Genetic Parameters*. Hayman [38] and Jinks [39] were used to determine the genetic and environmental components of variation and allied or related genetic factors in F_1_. The various components estimated were as follows:

(7) $$\begin{array}{c} E=\frac{\left\{\left(\text{Error SS}+\text{Rep}.\text{SS}\right)/\text{df}\right\}}{\text{ no}.\text{of replication}}=\text{expected environmental component of variance or error variance},\\ D={\text{V}}_{0\text{L}0}–E=\text{additive genetic variance},\\ F=2\;V_{0\text{L}0}–4\;W_{0\text{L}01}–2\frac{\left(n-2\right)E}{n}=\text{mean of Fr across arrays}, \end{array}$$

where Fr is the covariance of additive and dominant effects in a single array.

(8) $$\begin{array}{c} H_{1}=V_{0\text{L}0}–4W_{0\text{L}01}+4V_{1\text{L}1}–\frac{\left(3n-2\right)E}{n}=\text{dominance variance},\\ H_{2}=4V_{1\text{L}1}–4V_{0\text{L}1}–2E=H_{1}\left[1–\left(U-V\right)2\right], \end{array}$$

where *U* and *V* represent the percentage of positive and negative genes in the parents, respectively.

(9) $$\begin{array}{c} h^{2}=\frac{4{\left(M_{\text{L}1}–M_{\text{L}0}\right)}^{2}–4\left(n-1\right)E}{n^{2}}=\text{dominance effect}, \end{array}$$

as algebraic sum over the loci in heterozygous phase in the crosses.

*V*_0L0_ is the variance of parents, *V*_1L1_ is the mean variance of the arrays, *W*_0L01_ is the mean covariance between parents and arrays, *V*_0L1_ is the variance of the means of arrays, (*M*_L1_–*M*_L0_)^2^ is the difference between the parents' mean, and the *n*^2^ progenies' mean is known as the dominance relationship.

The estimation of genetic ratios was as follows: (H_1_/D)^1/2^ = the average level of dominance across all loci. The ratio of dominat to recessive allele were categorized as 0 = no dominance, >0<1 = partial dominance, 1 = complete dominance, and >1 = over dominance is indicated.

(10) $$\begin{array}{c} \text{Ratio dominant to recessive allele}=\frac{{\text{h}}^{2}}{{\text{H}}_{1}}. \end{array}$$

In the analysis of combining ability, the variance components in the ANOVA table were used to estimate broad-sense and narrow-sense heritability. The following equation was used to determine the heritability estimates for each kenaf attribute in each environment suggested by Rojas and Sprague [40]:

Broad-sense heritability:

(11) $$\begin{array}{c} h^{2}B=\left(\frac{V_{G}}{\text{VP}}\right)\times 100. \end{array}$$

Narrow-sense heritability:

(12) $$\begin{array}{c} h^{2}N=\left(\frac{V_{A}}{\text{VP}}\right)\times 100. \end{array}$$

*(5)1. Vr-Wr Graph*. The Vr-Wr graph can be used to interpret a diallel cross [32, 39]. By calculating the array variance (Vr), parent-offspring covariance (Wr), and regression of Wr on Vr, it is possible to test the adequacy of the simple additive dominance genetic model, discern the relative proportion of dominant and recessive alleles present in the common parents of each array, and determine the average level of dominance.

The Wri (covariance) values for each array were calculated by using the following formula:

(13) $$\begin{array}{c} \text{Wri}={\left(\text{Vri}\times V_{0\text{L}0}\right)}^{1/2}, \end{array}$$

where Vri is the variance of *r*^th^ array and *V*_0L0_ is the variance of parents.

By plotting Wr values against Vr values, the external limits of the parabola were found. The limiting parabola is drawn using the Wri values. The expected values of Wrei are needed to create a regression line. The following formula is used to acquire these Wrei values for each array separately:

(14) $$\begin{array}{c} \text{Wrei}=\text{Wr}-\text{bVr}+\text{bVri}, \end{array}$$

where Wr is the array means of covariances, Vr is the array means of variances, Vri is the individual array variance, and *b* is the regression coefficient.

*(5)2. Inference from the Vr-Wr Graph*. The average degree of dominance is shown by the regression line's position on the Vr-Wr graph. When the regression line crosses across the origin, it indicates complete dominance (*D* = *H*_1_). Partial dominance (*D* > *H*_1_) exists when the regression line crosses the Wr axis above the origin. It represents the absence of dominance when it crosses the origin, cuts the Wr axis, and contacts the limiting parabola. Overdominance is shown when the regression line crosses the Vr axis below the origin.

The order of dominance for each parent is determined by the position of parental points on the regression line. Parents with more dominant genes are closer to their ancestors than parents with more recessive genes. Parents with the equal amount of dominant and recessive genes fall in the middle of the range.

## 3. Results and Discussion

### 3.1. Variation in Pooled Environments across All Genotypes

All traits tested had highly significant (*p* ≤ 0.01) variations in environment and genotypes (parents and offspring) except for the middle and top diameters (Table 3). In the pooled quantitative data of the two environments for nine parents and their crosses, genotypes by environment interaction (*G* × *E*) were significant (*p* ≤ 0.01 or 0.05) except for plant height. The CV percent for fibre yield components varies from 12.02 to 69.05, indicating a wide range of variability in the features studied.

The genotypes performed differently across the environments studied, based on the results of the analysis on the combined data. These results indicate that the effects of genes controlling these traits were expressed differently in different environments. Most of the traits showed significant differences across genotypes and replications in the combined analyses of variance, indicating that the materials had enough genetic variation to improve these traits. The crop's extensive variation in botanical and agromorphological properties suggests that the genotypes are genetically diverse [41]. For all studied traits, the most significant environments, mean squares, and their interactions with genotypes were detected, indicating that the environment had enough seasonal variability to cause fluctuations in all population component rankings, i.e., different genotypes ranked differently from environment to environment. Alza and Fernandez-Martinez [42] and Abd El-Satar et al. [30] reported similar findings.

### 3.2. Genotype Performance Averaged over Two Environments

Table 4 shows the average comparison between all genotypes. The heights of the plants ranged from 230.44 to 280.59 cm, with parent P_4_ having the highest mean value and P_2_ having the lowest. The base diameters ranged from 20.88 mm (P_6_) to 25.74 mm (P_1_), with P_4_ having the highest core diameter (21.88 mm) and P_6_ having the lowest (17.15 mm). The middle diameter ranged from 9.69 mm (P_2_) to 12.49 mm (P_3_), with P_2_ and P_9_ having the largest and lowest top diameters, respectively. The most nodes were found in P_5_ (7.38), whereas the fewest were found in P_2_ (4.15). The period it takes to achieve 50 percent flowering varied between 55.67 and 69.17 days. The hybrid P_7_ was the first to reach maturity, while the parent P_2_ was the last (Table 4). In addition to other fibre yield components, Golam et al. [43] discovered that 50 percent flowering and days to maturity may be the two most relevant traits in identifying kenaf accessions.

The highest and lowest fresh stem weight was recorded for P_4_ (351.42 g) and P_6_ (222.41 g), respectively. The stick weights varied from 71.35 g (P_6_) to 123.24 g (P_3_). Among the parental lines, P_1_ had the highest mean fibre weight per plant (26.71 g), followed by P_4_ (24.78 g) and P_8_ (23.59 g), and P_2_ had the lowest (17.55 g) (Table 4). According to the findings, environmental factors influenced the expression of kenaf genetic characteristics. Parent P_3_ had the highest mean stick weight per plant (123.24 g), followed by P_7_ (96.26 g) and P_8_ (95.06 g). However, as the node number increased, the fibre production per plant decreased.

### 3.3. Combining Ability Analysis of Kenaf Hybrids for Fibre Yield Components

#### 3.3.1. Combining Ability Effects by Griffing's Method

For all analyzed traits in the combined data (Table 5) for both environments, analysis of variance for combining ability was performed using Griffing's technique [34]. GCA is well understood to be a function of additive gene effects and additive sections of epistatic variance. In contrast, SCA is a function of nonadditive gene effects and the remaining epistatic variance [44]. GCA effects were highly significant for all parameters measured except top diameter, according to the agronomic performance of the parental inbred lines. The SCA effects of the parental inbred lines were highly significant (*p* ≤ 0.01) for all traits except plant height and top diameter and significant (*p* ≤ 0.05) for days to 50 percent flowering, showing that additive gene effects predominantly influenced variability between those traits. In contrast, both additive and nonadditive gene effects controlled plant height and top diameter variations. The interaction of GCA with environment effects was highly significant (*p* ≤ 0.01) for all traits except plant height and fresh stem weight and significant (*p* ≤ 0.05) for base diameter and days to 50 percent flowering, indicating that the inbred lines' GCA was influenced by the environmental conditions surrounding the hybrids.

On the other hand, the SCA by environment interaction effects were highly significant for all traits except plant height and top diameter, while significant for days to 50 percent flowering, indicating that environments altered the effects of specific hybrid combinations on the expression of these traits. As a result, the impacts of nonadditive genes on phenotypes interacted with the environment more. For all traits except top diameter and node number, the mean squares of GCA were bigger than the mean squares of SCA, indicating that additive gene action predominated in our study across both environments (Table 5). Furthermore, the Baker ratio for the examined traits was altered from 0.52 to 0.93, indicating that the additive effect plays a larger role in determining the traits.

The combining ability analysis revealed significant GCA for all traits except top diameter and significant SCA for all traits except plant height and top diameter from the combined data of the two environments. The results indicate that variations for those traits were controlled mainly by additive gene effects, while both additive and nonadditive gene effects controlled variations for plant height and top diameter. For quantitative traits, the predominance of additive over nonadditive gene effects was rather prevalent. Similar findings were found in kenaf by Jianmin et al. [45] and Heliyanto et al. [46]. The analyzed traits (except top diameter and node number) had large mean square GCA values, implying that the parental materials examined had a high level of genetic variability. The investigated characteristics (except for plant height and fresh stem weight) significantly affected GCA by environment interaction, demonstrating that environmental variation influenced additive gene action. Likewise, the characters' environments had a significant impact on how they evolved [47]. Mostofa et al. [48] and Cai et al. [49] reported comparable outcomes in *Hibiscus cannabinus*, Sobhan [50] in *Hibiscus sabdariffa*, and Khatun [51] in *Corchorus capsularis* by combining ability studies.

For all traits except top diameter and node number, the mean squares of GCA were larger than SCA's, showing that additive gene action effects played a significant role in their inheritance. Although additive gene effects contributed the most to the variability in most traits, dominance and overdominance significantly influenced the genetic system that controls yield components. Because of the strong additive gene effects, this conclusion usually favours the breeding selection technique [52]. Mostofa et al. [53] discovered that fibre weight correlated with the presence of additive gene influences in the development of the characteristics. One dominant gene pair was responsible for days to first flowering and plant height, whereas three genes were responsible for raw fibre yield [54]. Pace et al. [18] discovered additive gene action to be more important for yield components such as plant height, fresh and dry bark weight, and usable stick in kenaf. Baker ratios [31] greater than 0.80 indicated that additive gene effects played a larger role in the genetic control of plant height, middle diameter, days to 50 percent flowering, fresh stem weight, stick weight, and fibre weight (Table 5). Additive influences contributed more to genetic variation, according to the Baker ratio. Additive variances are linked to heredity and react well to selection for improving desired traits. Anwar et al. [55] and Hassan et al. [56] both found similar findings in wheat research.

#### 3.3.2. General and Specific Combining Ability of Kenaf Genotypes for Fibre Yield Components

Plant height in a taller stature combination should have a positive GCA effect, while the node number should have a negative GCA effect [57]. In the pooled data (Table 6), the parent P_7_ had the maximum plant height (12.89) and the lowest negative GCA values for node number (-0.42), suggesting that they were strong general combiners for quality fibre yield and might be exploited in future breeding attempts. Parent P_4_ showed the most favourably significant GCA effect (*p* < 0.01) of plant height (9.11), base diameter (2.16), core diameter (1.93), middle diameter (0.70), fresh stem weight (54.54), stick weight (14.14), and fibre weight (3.02). With the exception of the P_4_, parent P_1_ (1.49) and P_3_ (1.38) were also positive and significant general combiners (*p* < 0.01) for fibre weight, with parent P_3_ having a positive and very significant GCA effect (*p* < 0.01) for stick weight (12.66). The parent P_2_ had the most positively significant GCA effect (*p* < 0.01) for days to 50 percent flowering (2.99). Parent P_7_ had the smallest GCA effect for days to 50 percent flowering (-3.89). The parent P_2_ showed the lowest negative and highly significant GCA effect (*p* < 0.01) for plant height (-16.83). For base diameter (-1.08), core diameter (-1.01), and fresh stem weight (-21.63), the parent P_9_ exhibited the lowest negative GCA values.

The effects of SCA on hybrids in various contexts in pooled environments are shown in Table 6. SCA impacts were seen in all 36 hybrids investigated, with 20 showing positive (desired direction) SCA influences on plant height. The hybrids with significant and beneficial SCA effects (*p* < 0.05) were produced by the cross P_5_ × P_8_ (23.63), which was evaluated as a good specific combiner for tallness, followed by P_7_ × P_9_ (19.91). The SCA effects on the base diameter ranged from -4.24 to 5.61. Of the 23 positive SCA effects, two (P_4_ × P_6_ and P_5_ × P_8_) were found to be highly significant (*p* < 0.01) for base diameter, while six (P_1_ × P_7_, P_2_ × P_3_, P_4_ × P_8_, P_4_ × P_9_, P_6_ × P_9_, and P_7_ × P_9_) were reported to be significant (*p* < 0.05). Six of the 36 cross combinations had significantly positive SCA values for kenaf core diameter, showing heterotic performance compared to their parents' mean. The cross P_2_ × P_3_ had the highest positive SCA effect (5.97), followed by P_4_ × P_6_, P_5_ × P_8_, and P_7_ × P_9_, indicating that they were found to be highly significant (*p* < 0.01) for the trait. The SCA effects for middle diameter stem varied from -1.52 to 2.97. The hybrid P_5_ × P_8_ had the best SCA effects, followed by P_7_ × P_9_ and P_8_ × P_9_, considered the best specific combiners for this trait. Another 18 crosses had positive but insignificant SCA values, implying that they could be used as average specific combiners. The SCA effects on top diameter ranged from -0.71 to 1.06. Three crossings exhibited significant positive SCA effects, P_4_ × P_7_, P_5_ × P_8_, and P_6_ × P_7_ were identified as good specific combiners for top diameter.

SCA impacts ranged from -1.94 to 2.52 for a given node number. Negative SCA effects were found in 14 crosses, with one (P_2_ × P_9_) being highly significant (-1.94) and another (P_1_ × P_9_) being significant (-1.31), showing that these hybrids had good SCA for lower branch stems and improved fibre yield. SCA impacts ranged from -5.55 to 4.91 days to 50 percent flowering. The significance of four of the 22 positive SCA effects was determined to be quite high. The cross P_2_ × P_5_ of the 15 positive SCA effects, which displayed a good specific combining capacity for this characteristic, had the most significant SCA effects. Five more crossings of the 23 positive SCA effects produced positive and significant SCA effects, making them good specific combiners for fresh stem weight. Three crosses, P_5_ × P_8_ (103.70), P_4_ × P_9_ (91.15), and P_2_ × P_3_ (78.19) had highly significant positive SCA effects, whereas two crosses, P_4_ × P_6_ (66.50) and P_7_ × P_9_ (58.02), had significant positive SCA effects, indicating that these hybrids had good SCA for fresh stem weight.

In kenaf, 23 cross pairings had positive SCA effects, while the other 13 had negative for stick weight per plant, SCA impacts ranging from -19.01 to 31.08. P_1_ × P_4_ had the largest significant positive SCA impact (31.08), followed by P_5_ × P_8_ (30.16), P_4_ × P_9_ (29.98), and P_7_ × P_9_ (29.54); it was an excellent specific combiner for the stick weight per plant attribute, meaning that it was a good specific combiner. Three crosses, P_2_ × P_3_ (11.70), P_4_ × P_9_ (5.31), and P_7_ × P_9_ (4.43), showed highly significant positive SCA effects for fibre weight, while four crosses, P_5_ × P_8_ (7.93), P_2_ × P_5_ (4.06), P_1_ × P_3_ (3.53), and P_1_ × P_7_ (3.45), showed significant positive SCA effects. This suggests that hybrids with higher fibre weights than their parents' means are the best specific combiners for increased kenaf fibre weight.

The research demonstrated that parents' GCA effects were linked to their crossings' SCA impacts, which had the highest significant positive intensity. Additive genetic variance is a major contributor to the GCA component. Because additive variance may be fixed, selecting qualities regulated by additive variance is a very effective strategy [58]. As a result, each parent's GCA variation has a major impact on the parents' decisions. A parent with a significant positive GCA effect is a good general combiner [59]. The high value of GCA for the traits of interest was dispersed across genotypes in this study, indicating that none of the genotypes employed had the best combination of GCA values for the various traits of interest. Parent P_4_ was among the best parental lines for plant height, base diameter, core diameter, middle diameter, fresh stem weight, stick weight, and fibre weight content, showing the accumulation of favourable additive genes for these traits in the hybrids. The parent P_3_ had favourable impacts on base diameter, fresh stem weight, stick weight, and fibre weight; on the other hand, the parent P_1_ was the greatest general combiner for fibre weight and contributed positively to the hybrid for these traits. The main purpose of this breeding effort is to create high-yielding hybrids with high potential fibre production comparable to or equal to existing cultivars.

Crosses with high vs. low general combiners outperform others in terms of yield components in general. According to an investigation of combining ability impacts [60], high-specific combiners involved high vs. high, high vs. average, high vs. low, average vs. average, average vs. low, and low vs. low combining parents. According to Jinks, overdominance and epistasis induced severe SCA effects in crosses with high vs. low and low general combiners [61]. In crosses comprising high vs. low general combiners for yield components, mutual cancellation of heterosis components, especially dominance and its association, resulted in unfavourable SCA effects [62]. Crossing two parents with low general combiners produces high performance, which is attributable to complementary gene action [63].

SCA effects were found to be significant for most yield traits, as mentioned in Table 6. For fibre weight, the hybrids P_2_ × P_3_, P_2_ × P_5_, P_4_ × P_9_, and P_7_ × P_9_ were shown to be the most effective specific combiners. The cross P_5_ × P_8_ is good for plant height, base diameter, middle diameter, top diameter, stick weight, and fibre weight. As the finest specific combiners for stick weight, the hybrids P_1_ × P_4_, P_5_ × P_8_, and P_7_ × P_9_ were selected. Days to 50 percent flowering for hybrids P_2_ × P_5_ were also picked as unique combiners.

Complementing gene effects could explain the strong SCA effects of these crosses. Crossings of P_1_ × P_4_, P_1_ × P_9_, P_2_ × P_3_, P_2_ × P_5_, P_4_ × P_6_, P_4_ × P_7_, P_4_ × P_9_, P_5_ × P_8_, and P_7_ × P_9_ showed promising heterotic responses and could be beneficial in future breeding program. Hybrid vigour can be induced by dominant, overdominant, or epistatic gene action in any combination of parents, according to Moll and Stuber [64]. In this study, both additive and nonadditive genetic components influenced fibre yield components, with nonadditive gene action dominating most of the characters.

#### 3.3.3. Hayman's Method of Analysis of Variance and Genetic Component Estimate

*(1) Hayman's Analysis of Variance*. After Morley Jones' modification [33], Hayman's ANOVA results for all tested traits in mixed environments were identical for additive effect (a), dominance effect (b), and error variance (Table 7). In combined environments, additive genetic effects (a) were significant for most of the traits, implying that both additive and dominance gene actions are involved in the inheritance of these traits. Most traits had a much larger a than b, indicating that additive effects were more important. For the top diameter and node number, a highly significant mean square was detected due to the interaction of b_l_ with the environment, and a significant mean square was detected for fibre weight and fresh stem weight, revealing that changes in soil types and climatic factors at each environment influenced the mean departure of the F_1_s from their mid-parental values for the two traits [30]. The remaining traits, on the other hand, exhibited negligible environment interaction mean squares with bi, indicating that they were stable in both environments. In addition, all traits had insignificant mean squares of b_2_ with environment interaction, indicating that b_2_ and variety heterosis components were consistent across the two environments [30]. All traits had insignificant mean squares of b_3_-environment interaction, demonstrating that b_3_ and specific heterosis components were stable across the two environments [30].

In combined environments, additive genetic effects (a) were significantly larger than dominance genetic effects (b), suggesting the presence of both additive and dominant gene actions in the inheritance of these traits [65]. The findings agree with those of Akter [66]. Xu [67] discovered a larger magnitude of the additive genetic variance than dominance in the inheritance of *Hibiscus cannabinus* flowering days. Significant mean square due to the interaction of b_l_ with the environment was detected for top diameter, node number, fibre weight, and fresh stem weight, indicating that the mean deviation of the F_1_s from their midparental values for the two traits was most likely influenced by differences in soil types and climate conditions at each environment [30].

*(2) Components of Variation and Genetic Parameters*. The genetic component analysis of Hayman [33] differed little from the analyses for additive (D), dominance (H_1_), and environmental error in most parameters (Table 8). The data were submitted to Hayman's diallel analysis [37] to isolate the genetic variance components and their ratios for all traits studied. For the eight examined traits out of 10, including fibre weight, positive dominance (H_1_ and H_2_) values were found, demonstrating the relevance of nonadditive components in the inheritance of these traits [68]. For the 10 traits, the magnitude of dominance (H_1_ and H_2_) was greater than the additive component (D), indicating overdominance [30]. H_1_ was greater than H_2_ for six of 10 traits, showing that the frequency of gene distribution in the parents was asymmetrical, implying dominance for all of these traits [30]. These findings matched with Akter [66], who observed that positive and negative alleles were not equal in the parents for all features in tossa jute. The *F* value was negative for all traits suggesting the presence of dominant recessive genes in the parents influencing these traits [30].

Because the balance of positive and negative alleles (UV parameter) is less than 0.25 for 6 out of the 10 traits and the b_2_ component is significant for a few, the frequency of dominant and recessive alleles cannot be assumed equal. Furthermore, the importance of nonadditive gene action in influencing the traits was demonstrated because h^2^_B_ and h^2^_N_ for the examined traits varied from 0 to 37.86 percent and 0 to 23.05 percent, respectively. Fibre weight shows a moderate heritability of 37.86 percent. Plant height had the highest narrow-sense heritability of 23.05 percent in the combined environment, followed by fresh stem weight (20.06 percent). On the other hand, poor narrow-sense heritability indicated that nonadditive gene effects predominated in trait inheritance [30]. The average degree of dominance at overall loci, as estimated by the (H_1_/D)^0.5^ ratio, was found to be greater than one for all traits except base diameter, core diameter, node number, and fibre weight, indicated by the Vr-Wr graph; overdominance gene effects have a role in the inheritance of most examined traits. On the other hand, the remaining attributes had a zero (H_1_/D)^0.5^ ratio, indicating no dominance in the control of the traits. Patil and Thombre [54] found a higher proportion of additive genetic components in kenaf in terms of days to flowering, plant height, and fibre length. The dominant impact is important for all traits except plant height, middle diameter, top diameter, and days to 50 percent flowering, according to the results of the ratio dominant to recessive alleles (h^2^/H_1_) (Table 8).

According to Hayman's analysis of variance and components of variation in gene action, all traits were influenced by both additive and dominant (nonadditive) gene actions. It also revealed that positive and negative alleles were not equally common in the parents and asymmetrical gene distribution at the loci, indicating that all these traits were dominant. However, the value of additive (D) and dominance (H_1_) of sqr (H_1_/D) were zero, which indicated the presence of no dominance effects for the base diameter, core diameter, node number, and fibre weight, while there were dominance effects for the other traits showing 1.34 to 2.06. Heritability in the narrow-sense assessed from genetic components appeared to be moderate to low for most traits. The moderate narrow-sense heritability values of plant height and fresh stem weight were indicative of their early generation selection success for breeding improvement [7]. All parameters of kenaf (fresh plant yield, defoliated stalk yield, plant height, basal diameter, middle diameter, and dry stalk yield) had a low narrow-sense heritability, according to Liu [7]. According to Falconer and Mackay [68], low additive effects and high dominant gene action caused reduction in narrow-sense heritability. Pedigree selection based on plant height and fresh stem weight can improve fibre yield.

### 3.4. Vr-Wr Regression Analysis

**Figure 1:**
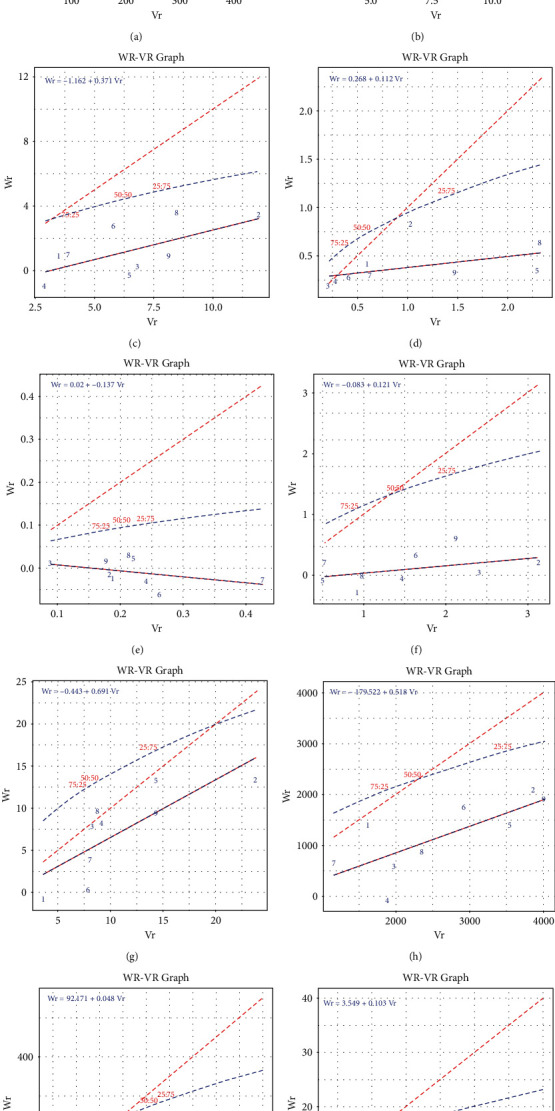
Wr-Vr graph for performing on (a) plant height, (b) base diameter, (c) core diameter, (d) middle diameter, (e) top diameter, (f) node number, (g) days to 50 percent flowering, (h) fresh stem weight (i) stick weight, and (j) fibre weight.

For the traits and their related statistics, Table 9 and Figure 1 show a graphical assessment of parent-offspring covariance (Wr) and array variance (Vr). The regression analysis showed that days to 50 percent flowering fitted with a simple additive dominance genetic model (Ho: *b* > 0 and Ho: *b* = 1) involving the nine parents studied. This is considerably different from zero but not from unity, showing no nonallelic interaction for these traits' inheritance. However, plant height and fresh stem weight showed highly significant “*b*” values in F_1_. It therefore did not follow the models clearly, which were significantly different both from zero and unity, indicating the presence of an interallelic interaction in the inheritance of these traits. The remaining of the nine traits in combined environments were considered to follow epistatic or nonallelic gene interaction as the regression coefficient significantly differed from unity but not from zero involving all nine parents studied.

The regression analysis revealed that days to 50 percent flowering differ considerably from zero but not from unity, indicating that nonallelic interactions do not inherit these traits. The findings were comparable to those of Sobhan [50], who found that in *Hibiscus sabdariffa*, a close relative of *H. cannabinus*, the days to flower suited the model well and the regression coefficient was substantially different from zero but not from unity. Plant height and fresh stem weight differed significantly both from zero and unity, indicating the presence of an inter-allelic interaction in the inheritance of these traits. The remaining seven traits in combined environments were considered to follow epistatic or nonallelic gene interaction as the regression coefficient significantly differed from unity but not from zero involving all nine parents studied. The regression coefficient *b* was not statistically significant when compared to zero. The deviation from unity for all 10 characters except for the top diameter indicated the validity of the hypothesis proposed by Hayman [62]. The nonsignificant *t*^2^ values satisfied the uniformity of covariance and variance (Wr-Vr) and thus supported the validation of the assumptions of Hayman [32] for these traits.

#### 3.4.1. Average Degree of Dominance

The deviation from the origin of the point where the regression line cuts the Wr axis, one of the information points acquired from the graph, measures the average level of dominance. In view of this, the intercept of the regression line on the covariance axis (Figures 1(b), 1(c), 1(f), 1(g), and 1(h)) is below the origin of base diameter, core diameter, node number, days to 50 percent flowering, and fresh stem weight, implying that these traits show overdominance. However, for plant height, middle diameter, stick weight, and fibre weight (Figures 1(a), 1(d), 1(i), and 1(j)), partial dominance was indicated by the regression line cutting the Wr axis above the place of origin. These findings match those of Vr-Wr regression analysis by Akter [66] for *Corchorus olitorius* and Khatun [51] for *Corchorus capsularis*.

#### 3.4.2. Parental Inheritance of Dominant and Recessive Genes

The array points along the regression line reflect the distribution of dominant and recessive genes between the parents. The parents with the most dominant genes are closest to the origin, whereas the parents with the most recessive genes are the furthest away. It is noticed that in Figures 1(a)–1(d) and 1(j), the parent P_4_ falls near the point of origin, in plant height, base diameter, core diameter, middle diameter, and fibre weight, as well as in Figures 1(d), 1(e), and 1(i), the parent P_3_ also falls near the point of origin, in middle diameter, top diameter, and stick weight. In Figures 1(b), 1(c), 1(g), and 1(f), the parent P_1_ also falls near the point of origin, in base diameter, core diameter, days to 50 percent flowering, and fibre weight. Results demonstrated the presence of dominant genes for the traits as mentioned earlier in the parents P_1_, P_3_, and P_4_. Those parents that are located closest to the regression line's start had more dominant genes for that trait, whereas those located farthest away had more recessive genes [69]. In contrast, the parents P_2_, P_8_, and P_9_ are the furthest from the origin in most features, implying that they have the most recessive genes for these traits.

### 3.5. Correlation among Fibre Yield Components

As demonstrated in Table 10, simple Pearson phenotypic correlation coefficients for fibre yield components were computed using proc corr SAS software version 9.4. Most traits do not exist in isolation; rather, they establish a complex connection with one another that ultimately determines the yield. The *r*-value of a correlation coefficient conveys the idea of a relationship between two unique traits by making a connection. Traits' correlation coefficients ranged from 0.05 to 0.99, indicating that the phenotypic variation is greater. Negative linear relationship, no linear relationship, and perfect positive linear relationship are shown by *r*-values of 1, 0, and +1, respectively. According to Field [70], values ranging from 0.5 to 1, 0.3 to 0.5, and 0 to 0.3 indicate strong, moderate, and low negative linear relationships, respectively, while values ranging from 0.5 to 1, 0.3 to 0.5, and 0 to 0.3 indicate strong, moderate, and low positive linear relationships, respectively.

Plant height, base diameter, core diameter, middle diameter, fresh stem weight, and stick/fibre weight all demonstrated a positive and highly significant correlation with dry fibre and stick weight. This was the most significant correlation yet observed for a single trait. For at least one of the types, in combined environments, a negative phenotypic correlation was seen in days to 50 percent flowering with fibre weight and stick weight. The fact that these attributes had a negative correlation with fibre weight suggested that they would not improve the trait. Plant height, base diameter, core diameter, middle diameter, node number, fresh stem weight, and stick weight all showed significant positive correlations with fibre weight, while top diameter and days to 50 percent flowering showed significant negative correlations. In contrast, there were significant correlations with all traits in the case of combined environment. Between the types, there was no discernible pattern or similarity. This suggested that progenies within each type may have a variety of strategies for expressing a certain phenotype. Significant and strong correlations will aid in selecting traits and qualities that influence commercial yield traits, making future selection easier and more reliable.

The negative relationship between fibre weight, stick weight, plant height, base diameter, core diameter, node number, and fresh stem weight suggests that early maturity reduces fibre yield (bast and core fibre) per plant [71]. Furthermore, the length of the growth environment may impact stem yield and other yield components like plant height. Early flowering plants have a shorter life cycle and produce less fibre than late flowering plants [69]. Several other significant correlations were also discovered between the various qualities investigated in this research. In the future, these correlations could be used to speed up the selection of superior progenies without quantifying all traits. The correlation analysis allows each trait's performance to be modelled with other major correlated traits, saving time and money in future investigations.

## 4. Conclusions

The fibre yield components evaluated in this study were genetically controlled by additive and nonadditive variants. From the combined data of the two environments, the combining ability analysis revealed strong GCA for all traits except top diameter and considerable SCA for all traits except plant height and top diameter. Additive gene effects mostly governed variations in those parameters. In contrast, according to the findings, plant height and top diameter were influenced by both additive and nonadditive gene effects. Except for top diameter and node number, GCA effects were higher than SCA effects, as shown by mean squares, indicating that additive gene action predominates for these traits. In conclusion, the parental lines P_1_ (ML5), P_3_ (ML36-10), and P_4_ (ML36-24) were outstanding general combiners for fibre yield components. In contrast, the hybrids P_1_ (ML5) × P_4_ (ML36-24), P_1_ (ML5) × P_9_ (ML36-21(2)), P_2_ (ML9) × P_3_ (ML36-10), P_2_ (ML9) × P_5_ (ML36-25), P_4_ (ML36-24) × P_6_ (ML36-27), P_4_ (ML36-24) × P_7_ (BJRI Kenaf4), P_4_ (ML36-24) × P_9_ (ML36-21(2)), P_5_ (ML36-25) × P_8_ (MLRing4P2), and P_7_ (BJRI Kenaf4) × P_9_ (ML36-21(2)) showed promising heterotic responses and could be beneficial in future breeding program. Plant height, middle diameter, days to first flowering, stick weight, and fibre weight indicated a clear partial dominance in the variance and covariance graphs. For most of the phenotypes evaluated, parents P_2_ (ML9), P_7_ (BJRI Kenaf4), and P_9_ (ML36-21(2)) had the most recessive genes, while parents P_1_ (ML5), P_3_ (ML36-10), and P_4_ (ML36-24) had the most dominant alleles. The Baker ratio showed that selection-based approaches highly responsive to additive effects enhance genetic improvement in the fibre output of hybridization-based breeding programmes. Because the fibre yield components studied have a high to moderate broad-sense heritability, a high Baker ratio, and moderate to low narrow-sense heritability, it was determined that selection should be done in advanced generations after homozygosity and genetically fixed.

## Figures and Tables

**Table 1:** Maximum and minimum temperatures, daily solar radiation, humidity, and average precipitation of the 2020/2021 season at University Putra Malaysia from planting to physiological maturity.

Month	Temperature (°C)		Daily solar radiation (MJ/m^2^/d)	Relative humidity (%)	Average precipitation (mm)
	Maximum	Minimum			
	1^st^ planting season (June–September 2020)				
June	28.87	23.41	16.15	86.63	245.03
July	28.53	23.07	17.39	86.91	310.55
August	29.09	23.30	21.01	84.93	101.57
September	28.62	22.99	16.77	86.36	244.04
Average	28.78	23.19	17.83	86.21	225.30
	2^nd^ planting season (March–June 2021)				
March	30.71	22.84	20.25	80.65	123.55
April	30.66	23.56	18.77	81.97	245.79
May	30.00	24.10	17.37	84.96	178.54
June	29.25	23.71	16.01	86.41	136.88
Average	30.15	23.55	18.10	83.50	171.19

Source: Agrobiodiversity & Environment Research Centre, MARDI, Selangor, Malaysia (2021).

**Table 2:** List of nine kenaf genotypes utilized as diallel cross parents.

Parent	Accession	Mutagenesis (GY) and generation	Source
P_1_	ML5	Acute (300), M_7_	Malaysian Nuclear Agency
P_2_	ML9	Acute (300), M_7_	Malaysian Nuclear Agency
P_3_	ML36-10	Acute (300), M_6_	Malaysian Nuclear Agency
P_4_	ML36-24	Acute (1300), M_6_	Malaysian Nuclear Agency
P_5_	ML36-25	Acute (1300), M_6_	Malaysian Nuclear Agency
P_6_	ML36-27	Acute (1300), M_6_	Malaysian Nuclear Agency
P_7_	BJRI Kenaf4	Conventional method, check	Bangladesh Jute Research Institute
P_8_	MLRing4 P2	Chronic, M_6_	Malaysian Nuclear Agency
P_9_	ML36-21(2)	Acute (800), M_6_	Malaysian Nuclear Agency

**Table 3:** Over two environments, a combined analysis of variance was performed for 10 fibre yield components of nine parents and their crosses.

Traits	Rep (Env)	Environment	Genotypes	*G* × *E*	Error	CV%
DF	4	1	44	44	176	
Plant height	1315.93	47672.79^∗∗^	1814.32^∗∗^	769.20	644.07	12.02
Base diameter	53.89^∗∗^	2644.69^∗∗^	45.51^∗∗^	28.09^∗∗^	7.80	20.39
Core diameter	60.42^∗∗^	2715.94^∗∗^	42.50^∗∗^	30.47^∗∗^	7.73	23.81
Middle diameter	128.33^∗∗^	3.95	7.08^∗∗^	4.75^∗∗^	2.18	19.19
Top diameter	42.75^∗∗^	534.35^∗∗^	1.24	1.59^∗∗^	0.92	44.68
Node number	2.27	5677.58^∗∗^	10.16^∗∗^	9.65^∗∗^	2.28	69.05
Days to 50 percent flowering	122.47^∗∗^	1946.76^∗∗^	85.63^∗∗^	59.30^∗∗^	33.12	12.55
Fresh stem weight	42044.66^∗∗^	471711.4^∗∗^	19213.8^∗∗^	10701.5^∗∗^	4361.16	30.88
Stick weight	3601.17^∗∗^	18465.42^∗∗^	2344.92^∗∗^	3071.60^∗∗^	877.24	36.86
Fibre weight	4.60	5968.42^∗∗^	126.22^∗∗^	102.29^∗∗^	17.80	33.59

Legend: ^∗^Significant at *p* ≤ 0.05 level. ^∗∗^Highly significant at *p* ≤ 0.01 level. CV: coefficient of variation; DF: degrees of freedom.

**Table 4:** Average performance of nine parents was evaluated for 10 fibre yield components in kenaf.

Traits	P_1_	P_2_	P_3_	P_4_	P_5_	P_6_	P_7_	P_8_	P_9_	$\bar{x}$ parents
PH	247.41 i-l	230.44 g-k	276.91 a-i	280.59 c-i	265.45 jkl	255.22 e-k	278.07 a-d	258.23 b-h	271.53 g-k	262.65
BD	25.74 g-l	21.44 e-k	24.70 a-e	25.47 a-f	24.61 i-m	20.88 g-m	22.36 c-i	21.40 e-k	23.33 k-p	23.33
CD	21.77 j-n	17.89 e-m	21.10 a-f	21.88 b-h	20.99 k-n	17.15 g-m	19.06 b-j	18.14 d-m	19.53 m-r	19.72
MD	12.17 c-j	9.69 f-k	12.49 b-f	12.29 b-f	12.23 ijk	10.95 d-j	10.85 d-j	11.57 c-j	10.73 jk	11.44
TD	4.31 b-h	4.47 gh	4.06 a-f	4.31 d-h	4.36 b-h	4.15 a-h	4.10 h	4.33 fgh	3.77 d-h	4.21
NN	6.91 h-l	4.15 e-l	4.80 abc	5.10 a-i	7.38 a-f	5.10 a-i	6.16 a-i	6.82 b-l	6.86 k-o	5.92
D50%F	60.67 b-j	69.17 c-k	57.67 c-k	63.83 d-n	58.00 b-i	64.00 b-j	55.67 f-n	60.50 c-m	55.83 a-f	60.59
FSW	328.89 a	225.00 ab	331.94 ab	351.42 abc	247.97 abc	222.41 abc	258.77 a-d	287.22 a-e	255.87 a-e	278.83
SW	93.78 g-o	74.56 f-o	123.24 a-i	90.62 a	79.60 d-o	71.35 c-n	96.26 c-m	95.06 c-m	81.46 i-o	89.55
FW	26.71 e-i	17.55 f-i	22.82 bcd	24.78 b-e	19.49 i-n	17.64 g-k	16.30 c-h	23.59 d-h	19.17 g-k	20.89

Legend: PH: plant height (cm); BD: base diameter (mm); CD: core diameter (mm); MD: middle diameter (mm); TD: top diameter (mm); NN: node number; D50%F: days to 50 percent flowering; FSW: fresh stem weight (g); SW: stick weight (g); FW: fibre weight (g).

**Table 5:** Mean squares in ANOVA of GCA and SCA across two environments for 10 fibre yield components from a 9 × 9 diallel cross (Griffing's method 2).

Traits	GCA	SCA	GCA × Env.	SCA × Env.	GCA/SCA	Baker ratio
Degrees of freedom (DF)	8	36	8	36		
Plant height	6114.71^∗∗^	858.68	761.26	770.97	7.12	0.93
Base diameter	69.61^∗∗^	40.15^∗∗^	19.77^∗^	29.94^∗∗^	1.73	0.78
Core diameter	57.06^∗∗^	39.26^∗∗^	22.34^∗∗^	32.28^∗∗^	1.45	0.74
Middle diameter	15.97^∗∗^	5.10^∗∗^	8.23^∗∗^	3.97^∗∗^	3.13	0.86
Top diameter	0.94	1.31	2.77^∗∗^	1.33	0.72	0.59
Node number	5.98^∗∗^	11.09^∗∗^	7.51^∗∗^	10.13^∗∗^	0.54	0.52
Days to 50 percent flowering	248.81^∗∗^	49.37^∗^	70.27^∗^	56.87^∗^	5.04	0.91
Fresh stem weight	45790.5^∗∗^	13307.9^∗∗^	4644.4	12047.5^∗∗^	3.44	0.87
Stick weight	5223.22^∗∗^	1705.30^∗∗^	4113.21^∗∗^	2840.14^∗∗^	3.06	0.86
Fibre weight	229.45^∗∗^	103.28^∗∗^	57.41^∗∗^	112.27^∗∗^	2.22	0.82

Legend: ^∗∗^Highly significant at *p* ≤ 0.01 level. ^∗^Significant at *p* ≤ 0.05 level.

**Table 6:** General and specific combining ability for 10 fibre yield components under tropical conditions.

Plant height									
	P_1_	P_2_	P_3_	P_4_	P_5_	P_6_	P_7_	P_8_	P_9_
P_1_	-9.44^∗∗^	10.53	4.60	-3.73	-24.32^∗^	-0.92	13.38	6.55	-2.07
P_2_		-16.83^∗∗^	-15.95	17.22	1.39	11.06	5.59	5.52	-26.96^∗∗^
P_3_			9.01^∗∗^	5.45	9.08	9.52	7.65	0.05	-1.59
P_4_				9.11^∗∗^	-4.08	-2.34	-8.70	3.84	4.23
P_5_					-0.41	12.88	-0.35	23.63^∗^	-14.17
P_6_						-2.33	-7.37	-5.02	-0.95
P_7_							12.89^∗∗^	1.91	19.91^∗^
P_8_								2.93	-4.60
P_9_									-4.94^∗∗^
Base diameter									
P_1_	0.52	0.50	2.05	0.54	-1.47	-0.05	2.27^∗^	0.28	-1.84
P_2_		-0.58^∗^	5.61^∗^	0.79	1.56	1.38	1.34	-0.49	-4.24^∗∗^
P_3_			0.81^∗∗^	0.44	-0.60	0.18	-1.35	0.65	-1.46
P_4_				2.16^∗∗^	1.10	3.12^∗∗^	-1.73	2.19^∗^	2.94^∗^
P_5_					0.06	-1.41	-0.24	5.54^∗∗^	-1.79
P_6_						-0.85^∗∗^	0.60	0.51	2.21^∗^
P_7_							-0.74^∗∗^	-1.36	4.49^∗^
P_8_								-0.29	0.40
P_9_									-1.08^∗∗^
Core diameter									
P_1_	0.41	0.22	1.95	0.52	-1.40	0.28	2.63^∗^	0.25	-1.90
P_2_		-0.51	5.97^∗∗^	0.84	1.85	1.25	0.91	-0.53	-3.87^∗∗^
P_3_			0.74^∗^	0.52	-0.51	0.08	-1.57	0.02	-1.28
P_4_				1.93^∗∗^	0.63	3.17^∗∗^	-1.87	1.89	2.71^∗^
P_5_					0.17	-1.14	-0.41	5.70^∗∗^	-1.56
P_6_						-0.84^∗∗^	0.84	0.28	2.03
P_7_							-0.61^∗∗^	-1.24	4.60^∗∗^
P_8_								-0.29	0.62
P_9_									-1.01^∗∗^
Middle diameter									
P_1_	-0.20	0.28	0.53	0.28	-1.52^∗∗^	0.16	-0.10	-0.05	-0.76
P_2_		-0.84^∗∗^	1.09	0.72	-0.23	0.32	0.05	0.01	-1.00
P_3_			0.35^∗^	0.06	-0.48	0.21	-0.08	-0.38	-0.56
P_4_				0.70^∗∗^	0.76	0.30	0.15	0.15	-0.23
P_5_					0.43^∗∗^	-0.26	0.44	2.97^∗∗^	-0.45
P_6_						-0.30^∗^	0.75	-1.22^∗^	0.60
P_7_							-0.11	-0.95	1.56^∗∗^
P_8_								0.36^∗^	1.73^∗∗^
P_9_									-0.40^∗^
Top diameter								
P_1_	-0.15	-0.30	0.69	-0.27	0.10	0.29	-0.71	-0.46	0.00
P_2_		-0.18	0.57	-0.20	-0.61	-0.41	-0.44	0.15	0.17
P_3_			0.03	-0.37	-0.03	-0.34	0.02	-0.23	0.30
P_4_				0.18	0.07	-0.40	0.78^∗^	0.62	0.46
P_5_					-0.06	-0.25	0.05	0.78^∗∗^	-0.45
P_6_						0.07	1.06^∗∗^	0.04	0.57
P_7_							0.01	-0.35	0.00
P_8_								0.13	-0.09
P_9_									-0.03
Node number									
	P_1_	P_2_	P_3_	P_4_	P_5_	P_6_	P_7_	P_8_	P_9_
P_1_	0.45^∗∗^	-0.28	1.48^∗^	0.62	0.56	0.76	1.14	-0.28	-1.31^∗^
P_2_		-0.37^∗^	2.42^∗∗^	1.53^∗^	1.64^∗∗^	1.06	0.30	0.23	-1.94^∗∗^
P_3_			-0.06	1.33^∗^	-0.65	-0.02	-0.32	1.32^∗^	-0.69
P_4_				0.04	0.25	0.98	-0.11	0.70	-0.64
P_5_					0.34^∗^	-0.55	-0.19	0.19	0.09
P_6_						-0.04	0.28	-0.66	2.52^∗∗^
P_7_							-0.42^∗∗^	-1.09	0.69
P_8_								0.24	1.64^∗∗^
P_9_									-0.16
Days to 50 percent flowering									
P_1_	1.07	-3.00	0.68	-3.23	1.83	-0.22	0.88	-0.75	4.30
P_2_		2.99^∗∗^	0.25	1.51	4.91^∗^	-4.14	-5.55^∗^	-2.17	-0.62
P_3_			-0.69	-1.31	1.59	-0.46	-3.20	0.35	1.56
P_4_				0.89	-0.15	-5.37^∗^	2.89	1.10	-2.02
P_5_					-0.17	-2.31	-1.55	-2.17	-1.29
P_6_						1.37^∗^	1.07	2.78	3.66
P_7_							-3.89^∗∗^	-1.29	-2.58
P_8_								-0.26	-2.37
P_9_									-1.31^∗^
Fresh stem weight									
P_1_	8.44	10.16	18.90	21.81	-50.67^∗^	9.36	30.51	-12.14	-0.16
P_2_		-15.98^∗^	78.19^∗∗^	21.65	23.50	49.34	22.19	-8.09	-59.07^∗^
P_3_			26.34^∗∗^	8.41	42.80	-19.09	14.80	-10.13	-40.61
P_4_				54.54^∗∗^	-1.61	66.50^∗^	-62.90^∗^	22.11	91.15^∗∗^
P_5_					-11.33	-20.13	28.80	103.70^∗∗^	-15.86
P_6_						-27.59^∗∗^	-13.46	-4.70	28.82
P_7_							-14.02^∗^	0.23	58.02^∗^
P_8_								1.22	-8.74
P_9_									-21.63^∗∗^
Stick weight									
P_1_	-0.19	1.33	1.93	31.08^∗∗^	-1.23	10.66	4.38	-1.64	-19.01
P_2_		-11.27^∗∗^	0.71	10.34	10.86	4.27	11.57	-3.98	-13.48
P_3_			12.66^∗∗^	-1.37	12.76	4.30	6.17	6.40	-10.92
P_4_				14.14^∗∗^	-1.93	22.74^∗^	-8.70	9.00	29.98^∗^
P_5_					-3.32	1.11	-11.93	30.16^∗∗^	3.53
P_6_						-10.32^∗∗^	-8.26	-9.90	6.88
P_7_							-0.43	-1.21	29.54^∗^
P_8_								3.05	9.04
P_9_									-4.33
Fibre weight									
P_1_	1.49^∗∗^	-1.20	3.53^∗^	1.69	-4.82^∗∗^	0.66	3.45	0.18	-0.66
P_2_		0.53	11.70^∗∗^	2.77	4.06^∗^	2.54	3.00	-0.20	-5.38^∗∗^
P_3_			1.38^∗∗^	0.39	0.06	-1.49	1.03	-2.18	-2.88
P_4_				3.02^∗∗^	1.29	2.89	-2.02	0.45	5.31^∗∗^
P_5_					-0.69	0.02	-2.05	7.93^∗^	2.04
P_6_						-2.17^∗∗^	0.05	-1.25	2.89
P_7_							-2.66^∗∗^	-0.86	4.43^∗∗^
P_8_								0.44	0.80
P_9_									-1.35^∗∗^

Legend: ^∗∗^Highly significant at *p* ≤ 0.01 level. ^∗^Significant at *p* ≤ 0.05 level. Estimates of general combined ability effects (gi) of each parent: P_1_, ML5; P_2_, ML9; P_3_, ML36-10; P_4_, ML36-24; P_5_, ML36-25; P_6_, ML36-27; P_7_, BJRI Kenaf4; P_8_, MLRing4 P2; and P_9_, ML36-21(2) and estimates of specific combining ability effects (Sij) of each cross for traits.

**Table 7:** Mean squares of Hayman and Jinks method for 10 traits of nine parents and their crosses over two environments.

Traits	a	b	b_1_	b_2_	b_3_	a × E	b × E	b_1_ × E	b_2_ × E	b_3_ × E
DF	8	36	1	8	27	8	36	1	8	27
PH	6114.71^∗∗^	858.68	2155.52	724.91	850.29	761.26	770.97	647.82	405.69	883.76
BD	69.61^∗^	40.15	428.65	18.66	32.13	19.77	29.94	112.99	14.89	31.32
CD	57.06	39.26	420.94	13.42	32.78	22.34	32.28	112.01	14.96	34.46
MD	15.97	5.10	19.58	2.63	5.30	8.23	3.97	0.16	2.24	4.62
TD	0.94	1.31	0.56	1.13^∗^	1.39	2.77	1.33	20.29^∗∗^	0.24	0.96
NN	5.98	11.09	141.60	8.22	7.11	7.51	10.13	96.75^∗∗^	9.53	7.10
D50%F	248.81^∗^	49.37	223.22	39.90	45.73	70.27	56.87	144.47	46.06	56.82
FSW	45790.5^∗∗^	13307.9	149535.8	4113.3	10986.7	4644.4	12047.5	57803^∗^	6091.2	12117.7
SW	5223.22	1705.30	22729.56	1136.09	1095.27	4113.21	2840.14	4472.14	1873.04	3066.24
FW	229.45^∗^	103.28	1213.06	45.53	79.29	57.41	112.27	474.76^∗^	52.58	116.53

Legend: ^∗∗^Highly significant at *p* ≤ 0.01 level. ^∗^Significant at *p* ≤ 0.05 level. a: additive variation; b: average square of domination; b_1_: average dominance; b_2_: symmetrical distribution of the alleles determining dominance; b_3_: residual dominance; E: environment; DF: degrees of freedom; PH: plant height (cm); BD: base diameter (mm); CD: core diameter (mm); MD: middle diameter (mm); TD: top diameter (mm); NN: node number; D50%F: days to 50 percent flowering; FSW: fresh stem weight (g); SW: stick weight (g); FW: fibre weight (g).

**Table 8:** Components of genetic variance [37] for all nine parents and their crosses over two environments for 10 traits studied.

Traits	Wr+Vr	E	D	F	H_1_	H_2_	UV	h^2^_B_	h^2^_N_	Sqr (H_1_/D)	h^2^/H_1_
PH	374.53	644.07^∗∗^	-369.83^∗∗^	-1025.68^∗∗^	-1161.29^∗∗^	-775.20^∗∗^	0.17	0	23.05	1.77	0.08
BD	8.14	7.80	-4.29	-10.58^∗∗^	3.45^∗∗^	6.56^∗∗^	0.48	29.28	14.41	0	8.20
CD	7.70	7.73	-4.56	-10.64^∗∗^	2.60^∗∗^	6.48^∗∗^	0.62	26.01	10.50	0	10.67
MD	1.42	2.18	-1.29	-3.15	-2.56	-1.28	0.12	0	13.35	1.41	-0.22
TD	0.21	0.92	-0.87	-1.29	-1.56	-1.03	0.16	0	0	1.34	0.21
NN	1.64	2.28	-0.95	-1.30	0.73	1.20	0.41	9.52	0	0	12.96
D50%F	18.14	33.12	-13.39^∗∗^	-40.67^∗∗^	-56.91^∗∗^	-37.47^∗∗^	0.16	0	14.16	2.06	-0.06
FSW	3766.76	4361.16^∗∗^	-2032.35^∗∗^	-6796.38^∗∗^	-4058.43^∗∗^	-1318.11^∗∗^	0.08	13.53	20.06	1.41	-2.27
SW	419.76	877.24^∗∗^	-633.49^∗∗^	-1306.31^∗∗^	-1372.45^∗∗^	-870.14^∗∗^	0.16	0	11.44	1.47	-0.96
FW	23.28	17.80^∗^	-4.33^∗∗^	-22.32^∗∗^	14.03^∗∗^	20.65^∗∗^	0.37	37.86	19.83	0	5.82

Legend: ^∗∗^Highly significant at the *p* ≤ 0.01 level. ^∗^Significant at the *p* ≤ 0.05 level. Wr+Vr: dominant effect; E: error variance; D: additive variance; F: relative frequency of dominant and recessive alleles in the parent; H_1_ - H_2_: dominance variance; UV: balance of positive and negative alleles; h^2^_B_ h^2^_N_: heritability of broad and narrow senses, respectively; Sqr (H_1_/D): mean degree of dominance; h^2^/H_1_: ratio dominant to recessive alleles; PH: plant height; BD: base diameter; CD: core diameter; MD: middle diameter; TD: top diameter; NN: node number; D50%F: days to 50 percent flowering; FSW: fresh stem weight; SW: stick weight; FW: fibre weight.

**Table 9:** Validity of Hayman and Jinks model for 10 traits of kenaf in a 9 × 9 diallel cross.

Traits	Regression coefficient	*b* = 0	*b* = 1	*t* ^2^
Plant height	0.414	3.109^∗^	4.400^∗∗^	6.994^∗∗^
Base diameter	0.339	1.997	3.892^∗∗^	4.046^∗∗^
Core diameter	0.371	2.258	3.822^∗∗^	4.183^∗∗^
Middle diameter	0.112	1.329	10.499^∗∗^	30.726^∗∗^
Top diameter	-0.137	-1.277	10.613^∗∗^	17.688^∗∗^
Node number	0.121	1.208	8.807^∗∗^	21.020^∗∗^
Days to 50 percent flowering	0.691	3.349^∗^	1.499	0.297
Fresh stem weight	0.518	3.040^∗^	2.826^∗^	2.398^∗^
Stick weight	0.048	0.357	7.012^∗∗^	10.246^∗∗^
Fibre weight	0.103	1.199	10.440^∗∗^	29.778^∗∗^

Legend: ^∗∗^Highly significant at *p* ≤ 0.01 level. ^∗^Significant at *p* ≤ 0.05 level. *t*^2^: test of validity of hypothesis.

**Table 10:** Combined analysis for correlation coefficient among 10 quantitative traits for nine kenaf parents and their crosses.

Traits	BD	CD	MD	TD	NN	D50%F	FSW	SW	FW
PH	0.56^∗∗^	0.56^∗∗^	0.33^∗∗^	-0.31^∗∗^	0.44^∗∗^	-0.52^∗∗^	0.60^∗∗^	0.41^∗∗^	0.55^∗∗^
BD		0.99^∗∗^	0.54^∗∗^	-0.29^∗∗^	0.74^∗∗^	-0.34^∗∗^	0.84^∗∗^	0.40^∗∗^	0.86^∗∗^
CD			0.54^∗∗^	-0.29^∗∗^	0.74^∗∗^	-0.35^∗∗^	0.83^∗∗^	0.40^∗∗^	0.86^∗∗^
MD				0.40^∗∗^	0.16^∗^	-0.16^∗∗^	0.58^∗∗^	0.48^∗∗^	0.38^∗∗^
TD					-0.67^∗∗^	0.32^∗∗^	-0.12^∗^	0.29^∗∗^	-0.36^∗∗^
NN						-0.40^∗∗^	0.53^∗∗^	-0.05	0.68^∗∗^
D50%F							-0.33^∗∗^	-0.20^∗∗^	-0.31^∗∗^
FSW								0.59^∗∗^	0.84^∗∗^
SW									0.46^∗∗^

Legend: ^∗∗^Highly significant at *p* ≤ 0.01 level. ^∗^Significant at *p* ≤ 0.05 level. PH: plant height; BD: base diameter; CD: core diameter; MD: middle diameter; TD: top diameter; NN: node number; D50%F: days to 50 percent flowering; FSW: fresh stem weight; SW: stick weight; FW: fibre weight.

## Data Availability

All data are provided in the results section of this manuscript.

## Supplementary Materials

Supplementary Materials Supplementary figure 1: the nine genotypes were mated in half diallel at Field 10 in University Putra Malaysia, excluding the reciprocals, to produce 36 F_1_ hybrids. Comparison of photographs showing stem pigmentation and leaf shape of parents and F_1_ population.
