# Supplementary material for: Estimating Genetic Analysis Using Half Diallel Cross Underlying Kenaf (Hibiscus cannabinus L.) Fibre Yield in Tropical Climates
Source: Biomed Res Int. 2022 Oct 19;2022:1532987. doi: 10.1155/2022/1532987 (PMC9605835; doi:10.1155/2022/1532987)
Supplement: Supplementary Materials — Supplementary figure 1: the nine genotypes were mated in half diallel at Field 10 in University Putra Malaysia, excluding the reciprocals, to produce 36 F1 hybrids. Comparison of photographs showing stem pigmentation and leaf shape of parents and F1 population. [file 1532987.f1.docx]

Supplementary figure 1: Comparison of photographs showing stem pigmentation and leaf shape of parents and F_1_ hybrids

| ♀ | ♂ | F_1_ | ♀ | ♂ | F_1_ |
| --- | --- | --- | --- | --- | --- |
| 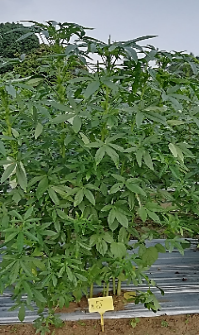 | 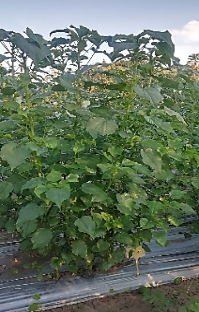 | 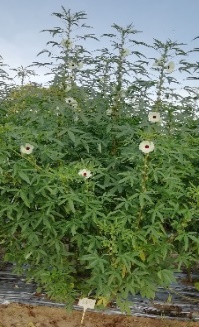 | 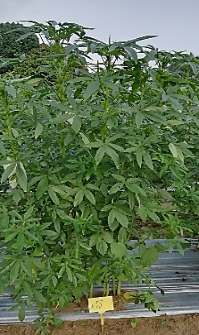 | 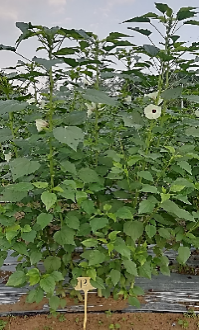 | 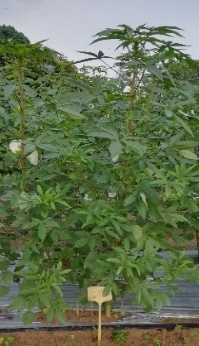 |
| P_1_ | P_2_ | P_1_ × P_2_ | P_1_ | P_3_ | P_1_ × P_3_ |
| 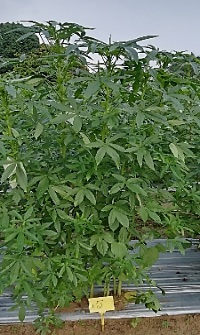 | 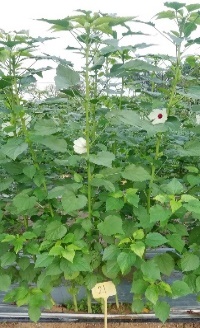 | 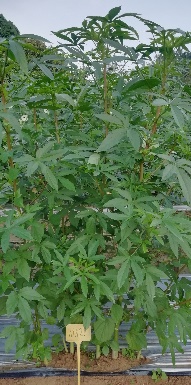 | 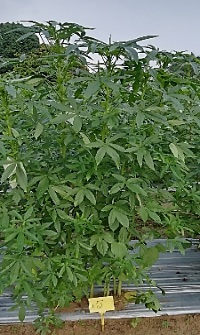 | 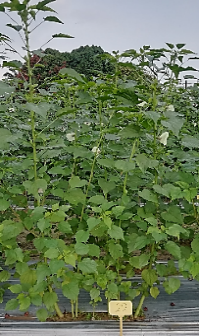 | 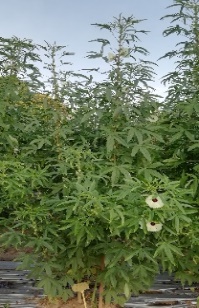 |
| P_1_ | P_4_ | P_1_ × P_4_ | P_1_ | P_5_ | P_1_ × P_5_ |
| 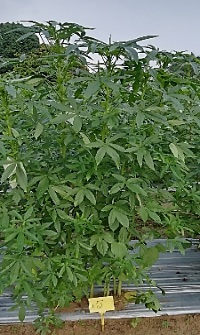 | 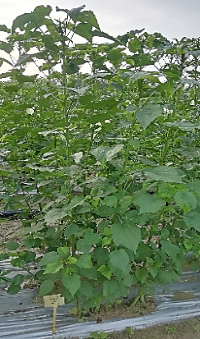 | 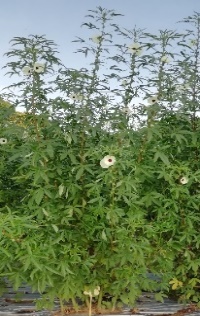 | 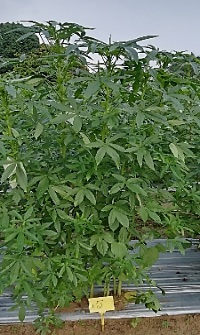 | 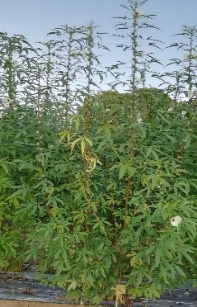 | 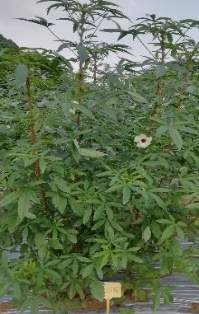 |
| P_1_ | P_6_ | P_1_ × P_6_ | P_1_ | P_7_ | P_1_ × P_7_ |
| 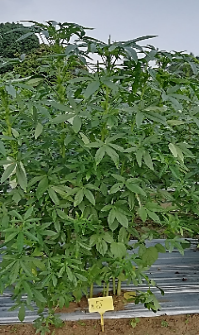 | 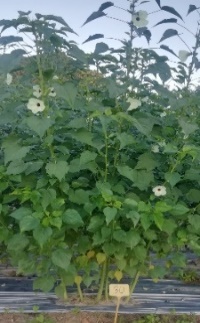 | 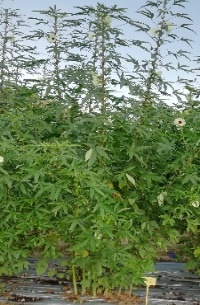 | 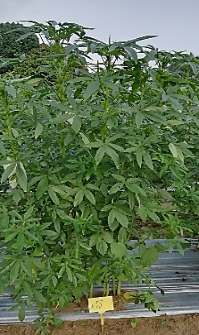 | 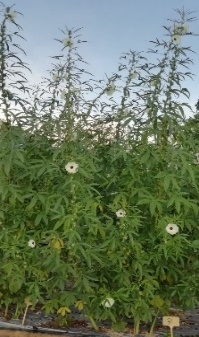 | 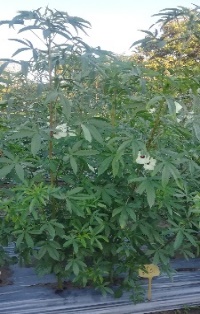 |
| P_1_ | P_8_ | P_1_ × P_8_ | P_1_ | P_9_ | P_1_ × P_9_ |
| 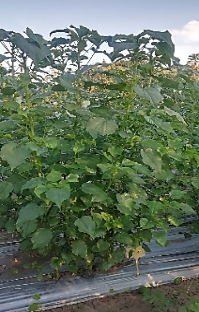 | 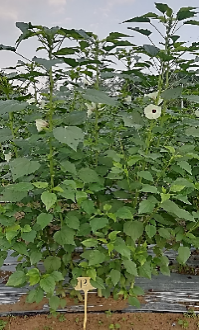 | 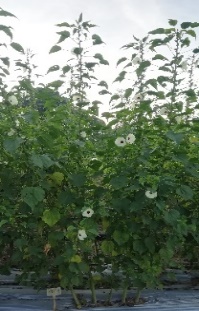 | 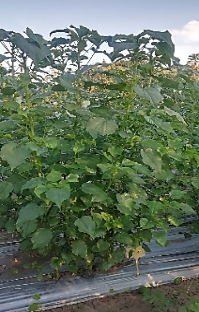 | 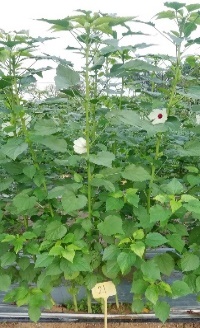 | 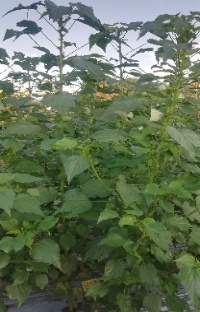 |
| P_2_ | P_3_ | P_2_ × P_3_ | P_2_ | P_4_ | P_2_ × P_4_ |

**Supplementary figure 1**: Continued

| ♀ | ♂ | F_1_ | ♀ | ♂ | F_1_ |
| --- | --- | --- | --- | --- | --- |
| 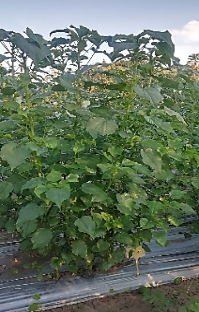 | 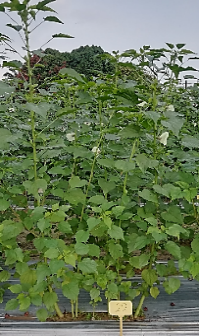 | 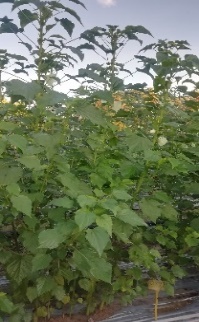 | 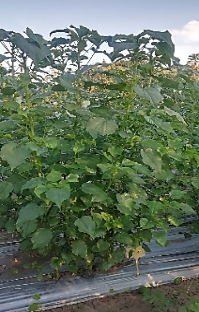 | 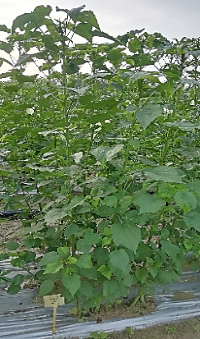 | 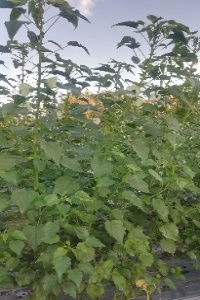 |
| P_2_ | P_5_ | P_2_ × P_5_ | P_2_ | P_6_ | P_2_ × P_6_ |
| 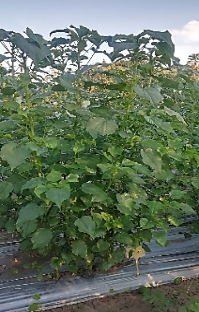 | 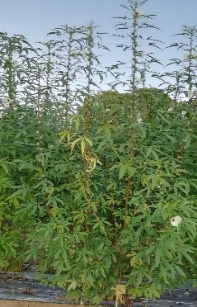 | 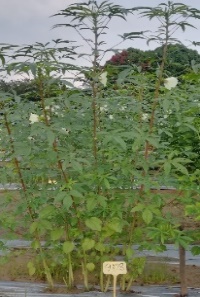 | 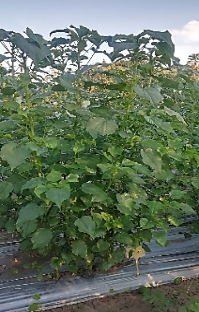 | 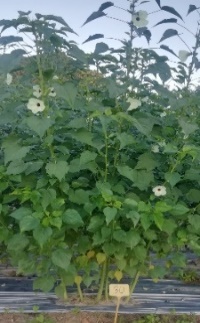 | 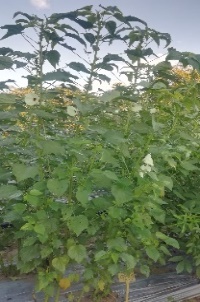 |
| P_2_ | P_7_ | P_2_ × P_7_ | P_2_ | P_8_ | P_2_ × P_8_ |
| 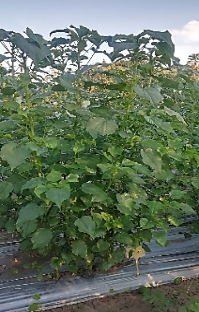 | 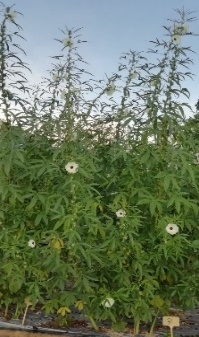 | 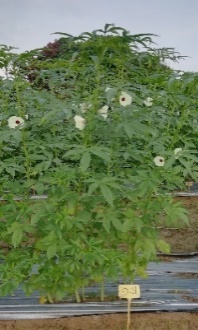 | 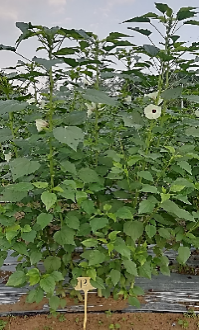 | 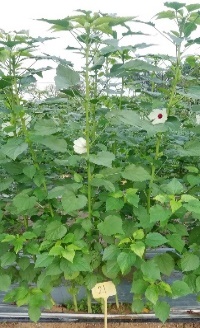 | 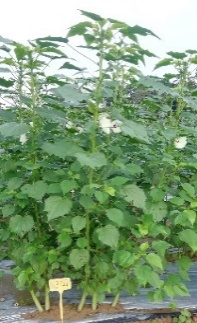 |
| P_2_ | P_9_ | P_2_ × P_9_ | P_3_ | P_4_ | P_3_ × P_4_ |
| 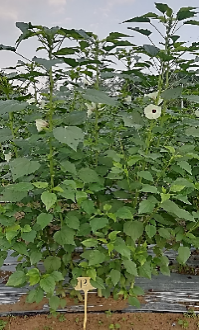 | 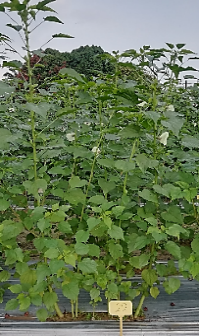 | 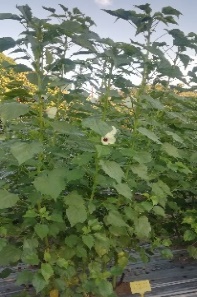 | 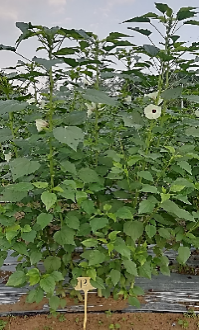 | 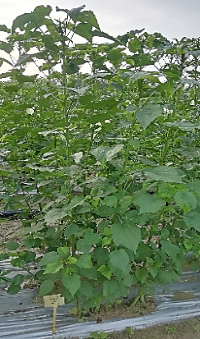 | 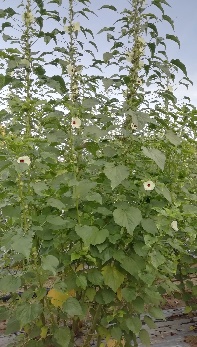 |
| P_3_ | P_5_ | P_3_ × P_5_ | P_3_ | P_6_ | P_3_ × P_6_ |
| 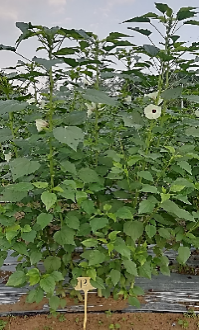 | 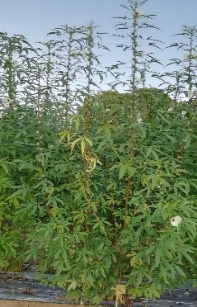 | 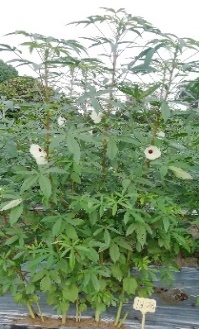 | 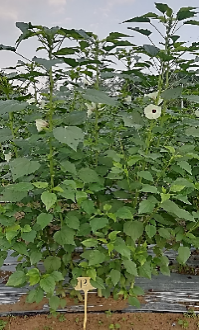 | 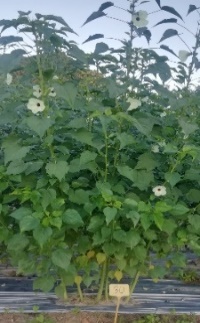 | 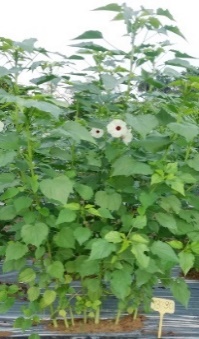 |
| P_3_ | P_7_ | P_3_ × P_7_ | P_3_ | P_8_ | P_3_ × P_8_ |

**Supplementary figure 1**: Continued

| ♀ | ♂ | F_1_ | ♀ | ♂ | F_1_ |
| --- | --- | --- | --- | --- | --- |
| 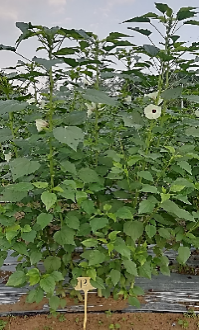 | 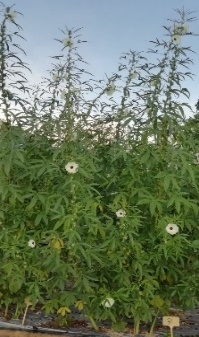 | 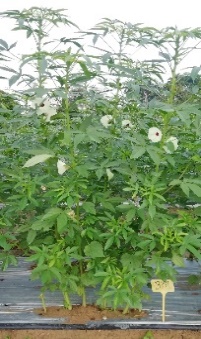 | 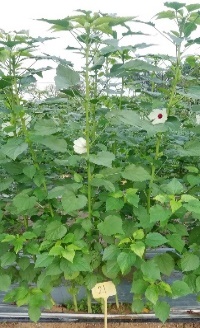 | 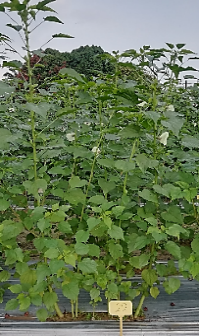 | 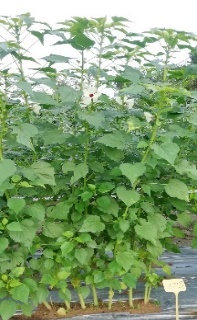 |
| P_3_ | P_9_ | P_3_ × P_9_ | P_4_ | P_5_ | P_4_ × P_5_ |
| 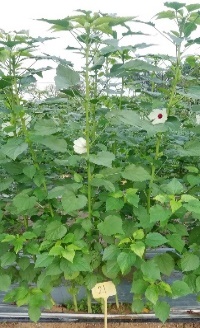 | 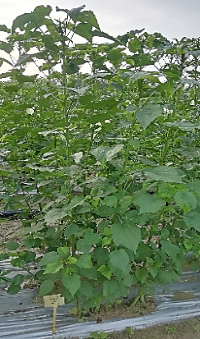 | 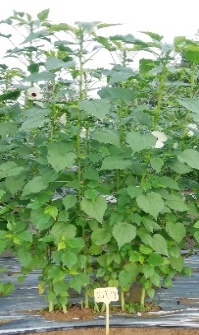 | 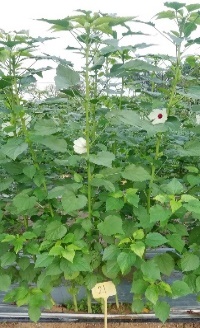 | 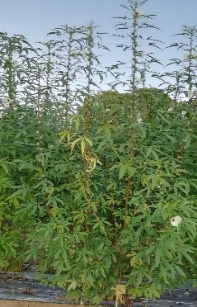 | 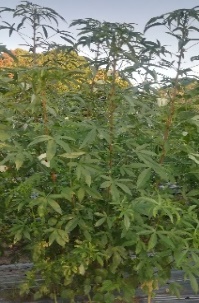 |
| P_4_ | P_6_ | P_4_ × P_6_ | P_4_ | P_7_ | P_4_ × P_7_ |
| 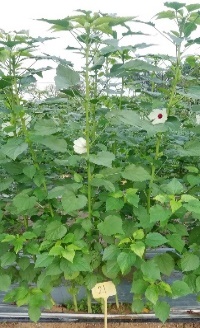 | 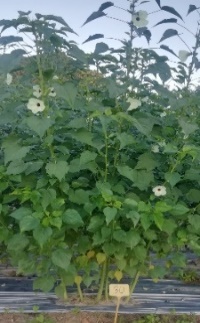 | 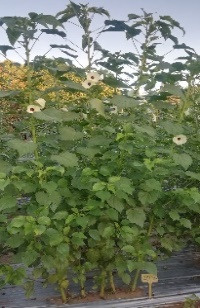 | 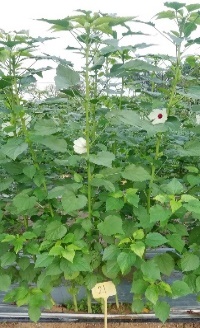 | 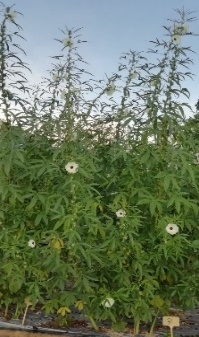 | 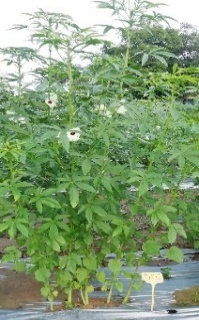 |
| P_4_ | P_8_ | P_4_ × P_8_ | P_4_ | P_9_ | P_4_ × P_9_ |
| 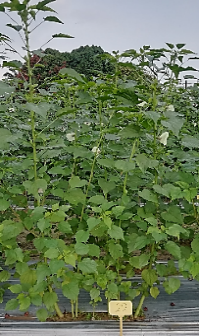 | 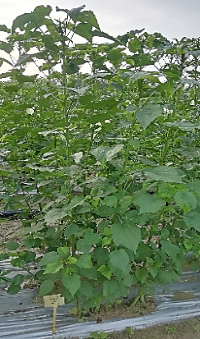 | 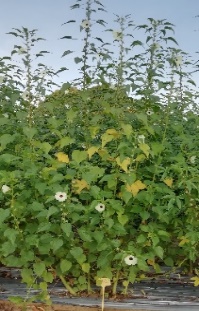 | 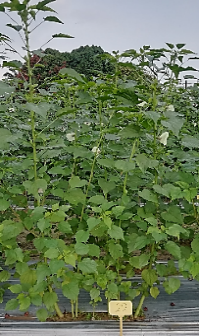 | 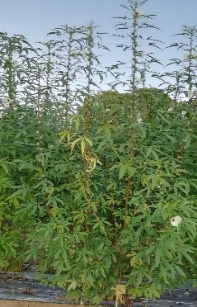 | 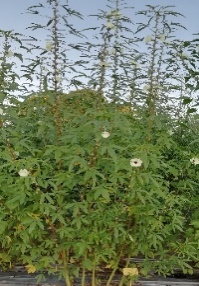 |
| P_5_ | P_6_ | P_5_ × P_6_ | P_5_ | P_7_ | P_5_ × P_7_ |
| 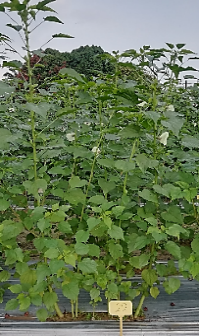 | 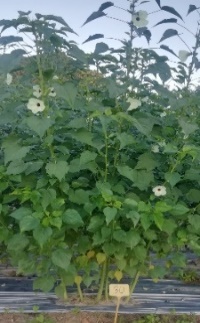 | 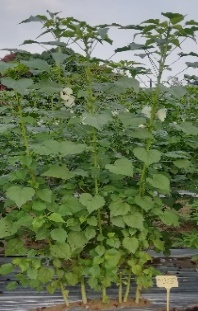 | 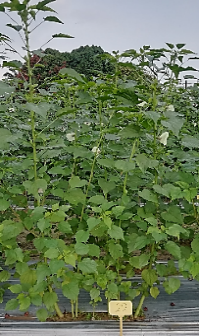 | 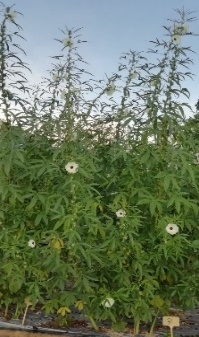 | 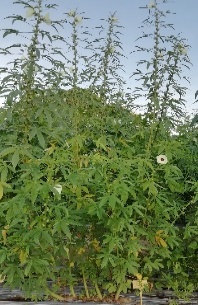 |
| P_5_ | P_8_ | P_5_ × P_8_ | P_5_ | P_9_ | P_5_ × P_9_ |

**Supplementary figure 1**: Continued

| ♀ | ♂ | F_1_ | ♀ | ♂ | F_1_ |
| --- | --- | --- | --- | --- | --- |
| 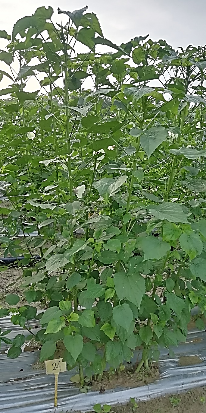 | 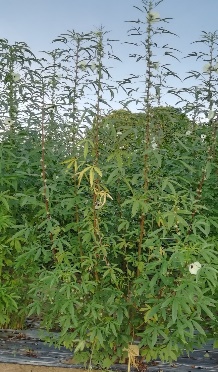 | 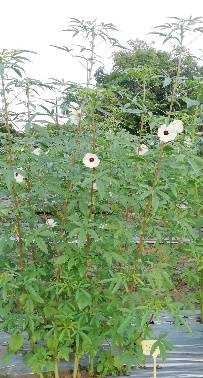 | 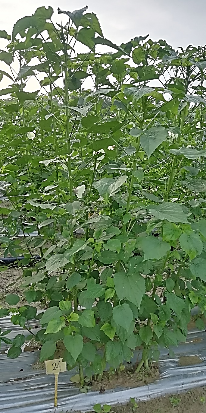 | 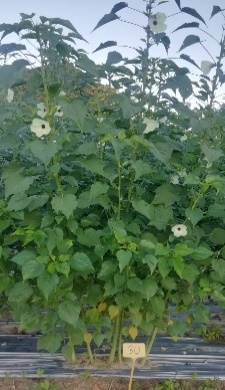 | 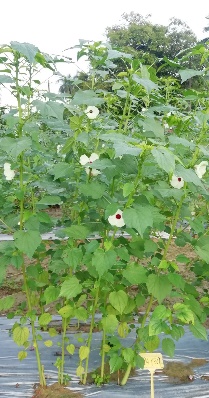 |
| P_6_ | P_7_ | P_6_ × P_7_ | P_6_ | P_8_ | P_6_ × P_8_ |
| 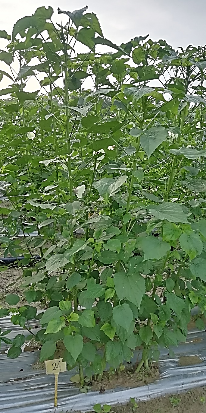 | 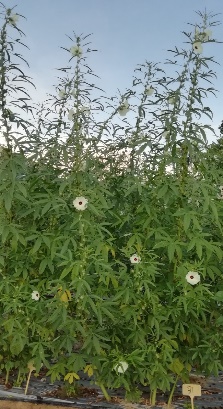 | 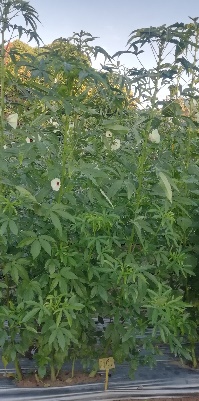 | 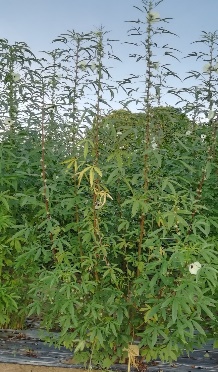 |  |  |
| P_6_ | P_9_ | P_6_ × P_9_ | P_7_ | P_8_ | P_7_ × P_8_ |
|  |  |  |  |  |  |
| P_7_ | P_9_ | P_7_ × P_9_ | P_8_ | P_9_ | P_8_ × P_9_ |
